# A Review on Manufacturing Processes of Biocomposites Based on Poly(α-Esters) and Bioactive Glass Fillers for Bone Regeneration

**DOI:** 10.3390/biomimetics8010081

**Published:** 2023-02-14

**Authors:** Xavier Lacambra-Andreu, Abderrahim Maazouz, Khalid Lamnawar, Jean-Marc Chenal

**Affiliations:** 1 CNRS, UMR 5223, Ingénierie des Matériaux Polymères, INSA Lyon, Université de Lyon, F-69621 Villeurbanne, France; 2 CNRS, UMR 5510, MATEIS, INSA-Lyon, Université de Lyon, F-69621 Villeurbanne, France; 3 Hassan II Academy of Science and Technology, Rabat 10100, Morocco

**Keywords:** manufacturing process, PLA, bioglass, mechanical properties

## Abstract

The incorporation of bioactive and biocompatible fillers improve the bone cell adhesion, proliferation and differentiation, thus facilitating new bone tissue formation upon implantation. During these last 20 years, those biocomposites have been explored for making complex geometry devices likes screws or 3D porous scaffolds for the repair of bone defects. This review provides an overview of the current development of manufacturing process with synthetic biodegradable poly(α-ester)s reinforced with bioactive fillers for bone tissue engineering applications. Firstly, the properties of poly(α-ester), bioactive fillers, as well as their composites will be defined. Then, the different works based on these biocomposites will be classified according to their manufacturing process. New processing techniques, particularly additive manufacturing processes, open up a new range of possibilities. These techniques have shown the possibility to customize bone implants for each patient and even create scaffolds with a complex structure similar to bone. At the end of this manuscript, a contextualization exercise will be performed to identify the main issues of process/resorbable biocomposites combination identified in the literature and especially for resorbable load-bearing applications.

## 1. Bone Regeneration: Application of Orthopedic Implants

In some cases, bone fracture needs a medical intervention to install an internal fixation. This system provides a temporary support to help the bone to restore the full function, ensure a correct alignment of fractured bones and minimize the possible complications during the healing. Furthermore, the device has to be biocompatible, inserted and removed without damaging the surrounding tissue and withstand dynamic loading forces without failure during the bone healing. 

Orthopedic implants are assigned principally as class II and class IIb for the (Food and Drug Administration of the U.S) and EMA (European Medicines Agency) respectively. However, they are considered as Class III when they exhibit an active function (e.g., incorporating medical products or induce biological effect).

### 1.1. Why This Review?

During these last years, the scientific community has shown an increasing interest on biodegradable synthetic polymer-ceramic materials [1,2,3,4,5,6,7] and the corresponding manufacturing process for bone tissue engineering [8,9]. The reviews of Boccaccini et al. [10] and Gritsch et al. [7] summarize the principal studies about synthetic biodegradable poly(α-ester)s and bioactive fillers of the last 20 years. Besides, the recent reviews of Dukle et al. [11], Palivela et al. [12] and Jain et al. [13] have presented the recent studies on additive manufacturing techniques for resorbable polyester and bioactive glass fillers. 

The aim of the present work is to inventory the studies of the different manufacturing techniques and final properties of biodegradable polyester filled with bioactive particles. First, the introduction includes a presentation of the different applications of these composites for internal bone fixation systems and the intrinsic properties of poly(α ester) and bioactive ceramics. Afterwards, we present a detailed review to highlight the different strategies to improve the final properties of implants of the recent studies of these composites structured by manufacturing process. A concluding section summarizes the current state of the field and highlights opportunities for further research.

### 1.2. Internal Bone Fixation

Bone plates, screw, nails or cages are use as internal fixation of fractures. Principally, internal bone fixation devices stabilize the bone from within the medullary canal (intramedullary nails) or fixed to the exterior of the bone (plates and strews).

As a function of geometry and position, plates can present five different functions: neutralization, compression, buttressing, tension band and bridging [14]. 

Compression plates apply a compression force to specific places to reduce the distance of bone fragments, increase fracture stability, and stimulate bone-to-bone interfaces. Neutralization plates protect the bone shear, bending and torsional forces. Buttress plates enhance the strength of weak cortical bone. In order to ensure a good loading force distribution, this kind of plate presents a large contact surface with a good bone-implant contact surface. 3D printing can design complex geometries to provide a good bone-implant contact surface and ensure a good loading force distribution. Tension band plates are placed to counteract the bending forces observed is some bearing bones. Installed in the tensile “side”, they convert the bending force to compression force. Bridge plates do not produce any force inside the injured zone and maintain the length, rotational and axial alignment.

Bone screws can also be used independently or combinate with plates or nails. They are the responsible of adjust the force transfer across the plate and fracture. The number and dimensions of screws to correctly install the plate depends on the health of bone (e.g., osteoporosis, osteonecrosis, infection), bone’s fragments and periprosthetic zone.

Besides, for intramedullary fixation (IF), nails are installed into the center of the bone with minimal surgical incision. They provide stabilization and act as a load-sharing device, allowing rapid rehabilitation after an injury. IF are widely used for rib fracture [15], forearms [16], tibia or femur fixation [17]. The materials for IF need to have the ability to be shaped according to the anatomical shape of the bone, be sufficient strength, and present an adequate stiffness that can meet the elasticity and compliance requirements of the anatomical region.

In the case of non-resorbable materials, these devices can be permanently implanted or removed once they are no longer required.

## 2. Composites: Why Poly(α-hydroxy Ester) and Bioactive Fillers?

### 2.1. Poly(α-hydroxy Ester)

Due to their excellent biocompatibility, resorption, sterilization ability, good mechanical and chemical stability under ambient conditions, ease of manufacture (thermal and solvent techniques) and control over the biodegradation rate, poly(α-hydroxy ester) have received considerable interest during the last 20 years. Poly(glycolic acid) (PGA) and Poly(ε-caprolactone) (PCL), the stereoisomers of polylactic acid (PLA), poly(L-lactic acid) (PLLA) and poly(D-lactic acid) (PDLA), and their copolymers (e.g., poly(D,L-lactic acid), PDLLA, and PLGA) are the most common synthetic resorbable polymers used for orthopedic devices.

**Figure 1:**
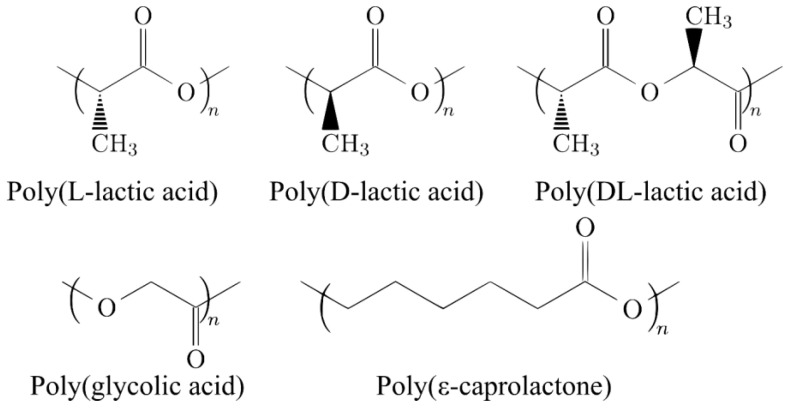
Chemical structure of the different poly(α-hydroxy ester).

These different poly(α-hydroxy ester) have been approved by FDA [18] and are already used for internal fixation devices. Figure 1 shows the chemical structure of the different poly(α-hydroxy ester). Each polyester presents a different degradation rate and medical applications [19].

PGA is frequently used as a material for biodegradable sutures, stents and orthopedic implants for tendon and cartilage repair since it presents a faster degradation kinetics than the others resorbable polyesters (less than 12 months) [20]. However, for some applications, the degradation takes place faster than expected and the medical device losses the mechanical properties and mass before the complete healing. 

The properties of PLLA, PDLA and PDLLA are highly dependent on the crystallinity, molecular weight and the ratio of D-lactide and L-lactide monomers for PDLLA [21]. They are extensively used in the orthopedic devices like plates, nails or screws [22]. Due to its crystalline phase, PLLA exhibits a similar modulus and tensile strength to PGA. Besides, the amorphous structure of PDLLA promotes elasticity, but decreases the tensile strength [19]. PLLA presents a long degradation rate (more than 3 years) whereas PDLLA shows a degradation time of 1–2 years due to the absence of crystallinity. 

Poly(ε-caprolactone) is a semi-crystalline polymer and presents a low degradation rate (2–4 years). Compared to PLA, PCL presents the advantages to be less hydrophilic and does not release acidic degradation products that could affect cell growth [3,23]. PCL is used principally for long-term implants as bone fixation or contraceptive devices [4]. However, the low degradation rate can even block bone ingrowth [4].

Poly(lactic-co-glycolic acid) (PGLA) is a copolymer composed of PGA and PLA. The principal advantages of PGLA copolymers are the possibility to adjust the degradation kinetics, mechanical properties and viscosity. 

Hence, the different degradation times and mechanical properties of bioresorbable polyesters provide a range of possibilities for biomedical applications.

The in-vivo degradation of poly(α-hydroxy ester) depends on different properties of the material, such as the nature of polymer, molecular weight, the geometry of the implant, porosity or processing technique [24]. In contact with body fluids, the poly(α-hydroxy ester) degradation is principally driven by a hydrolytic random chain scission [25,26]. Afterwards, when the molecular weight is close to critical molecular weight (Mc), polymer chains can diffuse through the body and through an enzymatic degradation process, the monomers and oligomers are assimilated by the body [25,27]. It has also been noted that the crystallinity of PLA tends to increase as polymer degrades. This can be attributed to the fact that hydrolytic chain cleavage proceeds preferentially in the amorphous regions, resulting in an increase in the polymer's global crystallinity [28]. 

However, internal fixation implants based on poly(α-hydroxy ester) exhibit a low affinity with body cells and, in some cases, the hydrolytic degradation can cause an inflammatory response of surrounding tissues [29].

It is also worth noting that the gamma-irradiation sterilization technique, the most widely used technique for orthopedic implants sterilization [30], increases the polymer matrix degradation [31]. 

### 2.2. Bioactive Fillers

Calcium phosphates, CaP, make up a family known as apatites have been widely used in orthopedic applications [32,33,34,35,36]. In general, the CaP used in medical devices are hydroxyapatite (HA), tricalcium phosphate (TCP), and a ratio of HA and TCP. Synthetic HA crystal is a bioactive and osteoconductive ceramic for which there exists long-term clinical experience. Although synthetic HA has the same composition to HA found in bone [37,38], the Ca/P ratio and specific surface are different (1.67 and 1.5 to 1.6 and 0.1 to 5 m^2^/g and 100 to 200 m^2^/g respectively). Besides, the low degradation rate, due to difference between natural and synthetic HA, causes inadequate degradation properties [39]. 

Tricalcium phosphates (α and β) have a higher solubility than HA [37]. They are also commonly used because of their biocompatibility, biodegradability, bioactivity and osteoconductivity [40,41]. Moreover, β-TCP offers the fastest in-vivo resorption rate among the commercial CaP. 

Biphasic CaP (HA and β-TCP) allow to control the degradation of orthopedic implant. The dissolution properties of a biocomposite with a filler of biphasic CaP are inversely proportional to the HA/TCP ratio [42].

**Figure 2:**
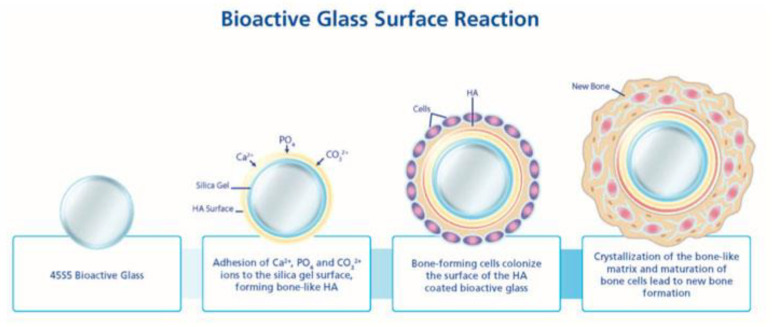
Bioactivity and bone enhancement steps of bioglass. Reproduced from [53].

Bioglasses (BG) are bioactive, osteoconductive and osteoinductive. They can be divided into three families: silicate (45S5, S53P4, 13–93), borate (13-93B3) and phosphate (CaP glass) [43]. After implantation, a hydroxyapatite (HA) layer is formed on the BG surface, followed by the attachment and proliferation of osteoblasts (Figure 2). Previous studies have demonstrated that, in a specific composition window of Na_2_O-CaO-SiO_2_, different glass compositions can present the ability to form a carbonated hydroxyapatite (cHA) layer, even if the kinetics are slowed down as compared to 45S5 BG (the most bioactive glass developed by Hench et al in 1971) [44,45,46,47]. Through the last years, the bioactive glass composition has been studied to control the bioactivity, resorption and mechanical properties [48,49,50,51].

However, the manufacturing process for orthopedic implants based on bioactive ceramics are time- and energy- consuming. Besides, these devices exhibit limited geometries, a mismatch in the mechanical properties of bone and ceramic implants as well as a brittle behavior mechanical response. Hence, the application of bioactive ceramics has been limited to non-loaded bone defects [52]. 

### 2.3. Poly(α-hydroxy Ester)/BG Composites

The combination of bioactive filler and a resorbable polymer allows to meet the mechanical and physiological demands of the host tissue [54]. The addition of CaP particles into a PLA matrix enhances biocompatibility, facilitates the integration of the implant in host tissues, and increases the modulus [33,55,56,57]. However, previous studies have reported some complications with β-TCP/PLA biocomposites because of the fast loss of mechanical strength over time [58]. Compared to CaP fillers, PLA/BG composites have shown in-vitro cHA formation on the surface and superior in-vivo bone regeneration [46,51,59,60]. Moreover, the inflammatory response due to the acidic degradation produced by the poly(α-ester) could be limited by the alkaline degradation of bioglass fillers [61,62,63]. 

Since these composites are biodegradable, the final properties are sensitive to the manufacturing process, bioactive filler nature and ratio, implant conservation, and implantation time [64,65,66]. In the case of poly(α-hydroxy ester)/BG composites, the thermal manufacturing processes exhibits an important effect on the molar mass reduction of the polymer matrix and on their final mechanical properties [64,65]. At high temperatures, a chemical reaction occurs between the silicate functions on the surface of BG and ester groups of the poly(α-hydroxy ester) accelerating the hydrolytic degradation [23,65,66]. Through the last years, several studies have tried to reduce this chemical reaction by coating the BG surface with a resorbable polymer [67,68], applying a thermal treatment on BG particles [66] or varying the BG composition [31,69], size and shape [66,70,71]. In order to avoid this hydrolytic degradation, numerous works proposed solvent casting manufacturing techniques to prepare poly(α-hydroxy ester)/BG composites [61,72,73,74]. However, since some toxic chemical substances may still be present in the final product, polymer solvents present significant obstacles to make the transition from laboratory to industrial application. 

Besides, in-vitro investigations into the degradation of poly(α-hydroxy ester)/BG composites have shown that BG particles accelerated the polymer matrix degradation when immersed in a PBS (Phosphate-Buffered Saline) solution [59,75,76,77,78,79]. These composites presented a significant loss of weight, molar mass, and mechanical strength from the first week of immersion. Therefore, it is essential to control the degradation effect of BG on the PDLLA during the manufacturing process and implantation in order to guarantee good mechanical properties throughout bone healing. 

In the following sections, we will present a detailed review of the different manufacturing techniques proposed in the literature to fabricate poly(α-hydroxy ester)/BG composites. The different mechanical, physical, morphological, microstructural, and bioactive properties will also be detailed.

## 3. Challenges and Opportunities

Following the criteria defined by the numerous studies of materials for orthopedic devices, the perfect material for orthopedic devices would be [43,80]:

- Biocompatible (no inflammatory response, immunogenicity, or cytotoxicity)
- Resorbable
- Bioactive (develop a cHA layer on its surface)
- Osteoconductive (have a structure that allows the formation of new bone)
- Osteoinductive (induce bone formation)
- Osteogenic (facilitate the formation of new bone)
- Radiolucent
- Easy to produce and with complex shapes
- With similar mechanical properties to those of cortical bone or sponge bone
- Easy to use surgically
- Sterilized (have an antibacterial surface to avoid possible infections)
- Hypoallergenic
- Can be used in a wide range of medical applications (trauma, fractures, bone infections, cancer...)

The recent research articles agree that resorbable materials, their design, and their manufacturing process are expected to improve the treatment of bone fractures [81]. To realize this new concept of bone plates, many attempts have been made by using different biomaterials and manufacturing process strategies. Hereafter, we present the current improvement strategies observed in the literature.

### 3.1. Therapeutic Applications/Therapeutic Release

**Figure 3:**
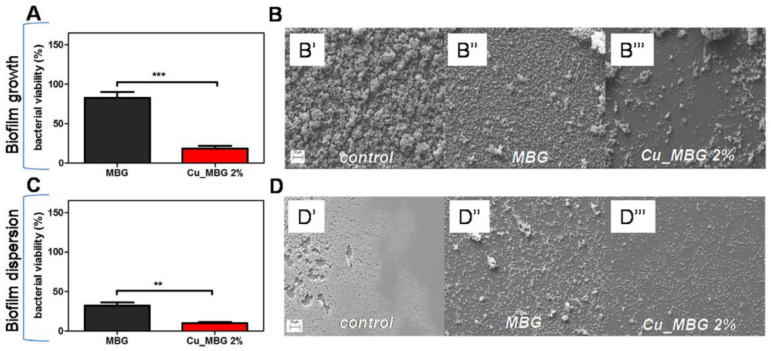
Bacterial viability and morphology of biofilms treated with MBG and Cu_MBG 2% suspensions. Both suspensions were added to S. epidermidis RP62A biofilm formation cultures (**A**) or after the formation of a stable staphylococcal biofilm (**C**). Bacterial viability is designed by *** and ** for (**A**) and (**C**) experiments respectively. SEM representative images of the staphylococcal biofilms formed in the absence (control) or in contact with both types of nanoparticles for 24 h are shown (**B**). SEM images of post formed biofilms, untreated (control) or treated with both MBG or Cu_MBG 2% nanoparticles for 24 h, are shown (**D**). Reproduced from [88].

One of the most serious complications associated with orthopedic implantations is bone implant-associated infection. Drug therapy has shown positive results for the treatment of bone defects [82]. In order to achieve local and targeted therapeutic effects, antibiotics, drugs or metallic ions can be introduced into the biomaterials used for medical devices [83]. The incorporation of metallic ions as strontium (Sr) [84], zinc (Zn) [85,86], magnesium (Mg) [87], copper (Cu) [88], silver (Ag) or cobalt (Co) [89] can improve the physicochemical and biological properties of BG composites. For example, Ag or Mg nanoparticles have a strong inhibitory and bactericidal effect, and Co ions interfere in physiological process such as oxygen transport in blood. Figure 3 exhibits the benefits of Cu ions to reduce the biofilm formation on the bone implants.

Several studies have shown the benefits of therapeutic release. For example, Vallet-Regí team developed biocomposites with a therapeutic release by introducing drugs, metallic ions, and antibiotics into poly(α-hydroxy ester)/BG composites to reduce the inflammatory response [90], the infection rate during implantation [86] and treat the osteoporosis [91] or bone cancer [92]. 

### 3.2. Scaffolds

3D printing techniques open up a new range of possibilities for bone tissue engineering. Among these opportunities, scaffold fabrication has been the main focus of researches in the domain of orthopedic systems.

The principal objective of scaffolds is to provide an environment in which bone formation is accelerated and can take place with no complications. These porous scaffolds facilitate adhesion, proliferation, differentiation, and migration of cells to facilitate tissue regeneration [93]. Usually, they present interconnected pores to facilitate the body fluid circulation, transportation of cells, and metabolic wastes. Moreover, the level of porosity and pore dimensions directly affects cell attachment, biodegradation, and drug release rates since the amount of scaffold/body interface surface are correlated. However, in the case of polymer-based scaffolds, excessive porosity affects mechanical performance and difficult their utilization when exposed to external loads. Therefore, several authors studied the optimization of porosity, pore sizes, and pore architecture to balance mechanical strength and bone formation [1,94,95].

Besides, 3D printing of resorbable metallic Mg- or Zn-based scaffolds fabricated by 3D printing are considered as an interesting strategy to combine the advantages of scaffold structure with the controllable reduction of mechanical properties to obtain similar performance of bone [96]. 

### 3.3. Shape Memory Effect (SME)

Shape memory effect (SME) presents the advantage of being able to be compressed into a temporarily smaller size and then return to their initial shape and size under an appropriate external stimulus (heating). Orthopedic implants with SME could reduce the size of the surgical incision area and hence, reduce the postoperative time and possible complications [97]. Recent studies have shown the possibility of poly(α-hydroxy ester) with bioactive fillers to enhance the biological performance and shape memory properties for internal fixation systems [98,99,100,101].

### 3.4. Functionally Graded Materials (FGM)

Functionally Graded Materials (FGM) are materials whose structure and/or composition gradually change in one or multiple directions. Therefore, the properties change in order to respond to specific requirements [102,103]. Osteochondral tissue is composed of the cortical bone, the cancellous bone, and the cartilage. Moreover, hierarchical structure porosity, and hierarchical composition (collagen, carbonated hydroxyapatite, and water) in bone makes the osteochondral tissue the perfect example of functionally graded material. 

**Figure 4:**
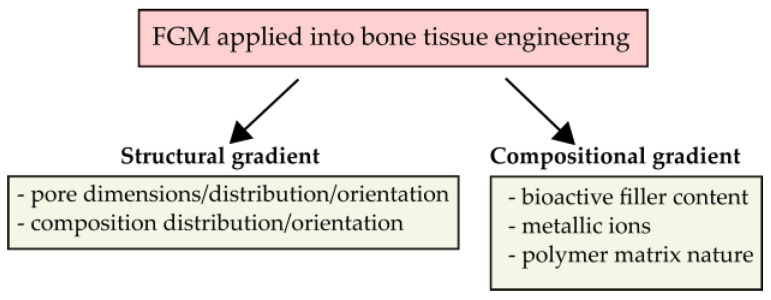
Diagram of the different FGM strategies for bone tissue engineering.

In bone tissue engineering, a functional gradient can contribute to obtain a suitable structural strength, porosity, bioactivity, or drug release [102,103,104] (Figure 4). The potential of resorbable poly(α-hydroxy ester)/bioactive fillers FGM composites for bone tissue engineering applications has been studied by different authors over the last years. 

A FGM strategy can be used to adjust the stiffness of orthopedic implants and bone replacements by using with controlled compositional gradient structure and/or a graded porous structure to mimic the mechanical response of bone and thus, avoid stress-shielding and reduce aseptic loosening [105,106,107]. Caridade et al. [108] and Pawlik et al. [109] developed FGM membranes with a bioactive and a barrier side to promote bone formation on one side and prevent it on the other side [108,109]. In parallel, Li et al. [110] fabricated a bi-layered membrane by a two-step method. The bi-layer membranes were composed of a dense and smooth layer to prevent the infiltration of connective tissue and a porous bioactive layer to promote osteogenesis. These systems are useful for bone repair in cranial, maxillofacial areas and in dental applications, where limited mechanical loading exists. Furthermore, other studies have combined different materials to provide bioactivity and enhance the mechanical properties [111,112,113].

## 4. Manufacturing Process

As presented in the previous sections, there are several materials and fabrication methods that could potentially meet the desired bone fixation requirements. However, not all materials and fabrication methods are compatible with each other, and the right combination should be selected when designing an orthopedic implant. The objective of this section is to present the different manufacturing techniques used to fabricate poly(α-hydroxy ester)/BG composites. The principal mechanical and microstructural properties of the discussed articles are resumed at the end of each section.

### 4.1. Solvent Route

The results of different studies presented in this section are summarized in Table 1.

#### 4.1.1. Solvent Casting

The solvent casting method is usually used in order to characterize the in vitro behavior of biocomposites of poly(α-hydroxy acids)/BG, fabricate pellets and films. Interestingly, the solvent casting technique prevents thermal degradation during processing [61,72]. Firstly, the resorbable polyester is dissolved in a solvent (usually with chloroform, acetone or DMC). Once dissolved, glass powder is mixed with solution until the particles are well dispersed. Afterwards, the polymer and glass blended solution is cast in a PTFE mold to facilitate solvent evaporation. Finally, the film is cut into a precise geometry or milled and sieved to obtain granules.

Navarro et al. [73] fabricated a composite based on P(L/DL)LA with a 95L/5DL ratio blended with a BG type G5 using chloroform as a solvent. The aim of their study was to compare the degradation response of PLA/BG composite with PLA. BG composite had a more complex degradation behavior than P(L/DL)LA. The hydrolytic degradation of PLA was accelerated by the fluid penetration in the polymer/BG interface induced by the partial BG dissolution. Moreover, the formation of a cHA layer on the surface and the buffering effect of BG increased the pH of the surrounding fluid. Aliaa et al. [114] used a solvent casting method by mixing 95% of PLA and 5% of PEG in 5 vol% of chloroform, and afterwards BG powder was added to obtain concentrations at 1 and 2.5 wt%. The apparition of voids within the PEG/PLA/BG films indicates its degradation. Moreover, they noted that suspensions with a non-homogeneous distribution lead to voids creation and weak interfacial bonding; causing a diminution of mechanical properties. Gao et al. [115] fabricated a PDLLA/bioglass film using different BG particles (45S5, mesoporous 58S, and 58S) through a solvent casting technique. Biocomposites films filled with surface-modified BG particles presented a better particle distribution in the matrix than non-surface-modified BG. Moreover, the modification did not significantly affect the bioactivity or the mechanical properties. Tamjid et al. [71] prepared a PCL composite film containing 5 wt% of BG particles at different size. The introduction of BG nanoparticles increased the elastic modulus and improved bioactivity. However, BG nanoparticles increased the hydrophily and consequently, the degradation kinetics. Terzopoulou et al. [116] fabricated PCL membranes with two types of BG (containing Sr or Ca ions). Moreover, the osteogenesis properties were enhanced by adding bisphosphonate drug ibandronate (IBA) into the composites. Pawlik et al. [109] fabricated a film of PCL/PLGA blend with BG particles biocomposite by solvent casting route. They observed that the ratio of PCL/PLGA and BG composition are two key factors to control the mechanical and bioactivity properties. Mohammadkhah et al. [117] studied the mechanical properties, bioactivity, and in-vitro degradation of biocomposites based on PCL filled with 45S5 and 13-93B3 BG. The 13-93B3 BG presented a higher degradation and bioactivity and a slight decrease of elastic modulus.

#### 4.1.2. Scaffolds Systems by Solvent Casting

During the last years, several studies fabricated porous PLA/BG composites to increase bone in-growth. Solvent techniques like solid-liquid phase separation method (SLPS), solvent casting particulate leaching (SCPL), and gas foaming (GF) can be used to fabricate porous PLA/BG composites. 

#### 4.1.3. Gas Foaming (GF)

Gas foaming is the process of forming porous structures by enabling gas flow or bubble formation inside a mixture of polymer solutions. The polymer is mixed with a solvent, a foaming agent, and a binder to create a polymer paste to be molded into a particular geometry. After a partial solidification, the chemical reaction of the foaming agent is activated to create the desired porous geometries. Besides, a second gas foaming technique consists in inject an inter gas like N_2_ or CO_2_ into the polymer solution to create an internal porous structure [1]. Song et al. [118] fabricated a highly interconnected PLGA/BG porous scaffold by CO_2_ foaming to enhance the biological fluid circulation. The authors ensured an interconnected porosity by controlling the pressure, venting foaming, and temperature. The addition of BG particles increased slightly the mechanical properties. Dong et al. [119] fabricated a composite foam based on PLGA filled with BG particles grafted with PLLA. The in-vivo tests confirmed the good biocompatibility, homogeneity, and mechanical properties of PLGA/g-BG foams.

#### 4.1.4. Solid-Liquid Phase Separation Method (SLPS) or Freeze-Drying 

The freeze-drying is the process of creating microporous structures by freezing a polymeric solution suspended in another liquid (e.g., water, camphene) to a lower temperature to create a phase separation between the freezing vehicle and the precursor solution [1]. Afterwards, the porous composite is produced by the melting and extraction of frozen vehicle crystals that have been trapped inside the polymerized gel [1]. Fabbri et al. [74] fabricated a high porous composite (around 90% of porosity) of PCL/BG 45S5 by solid-liquid phase separation method (SLPS) with a good cell proliferation during in-vitro tests.

**Figure 5:**
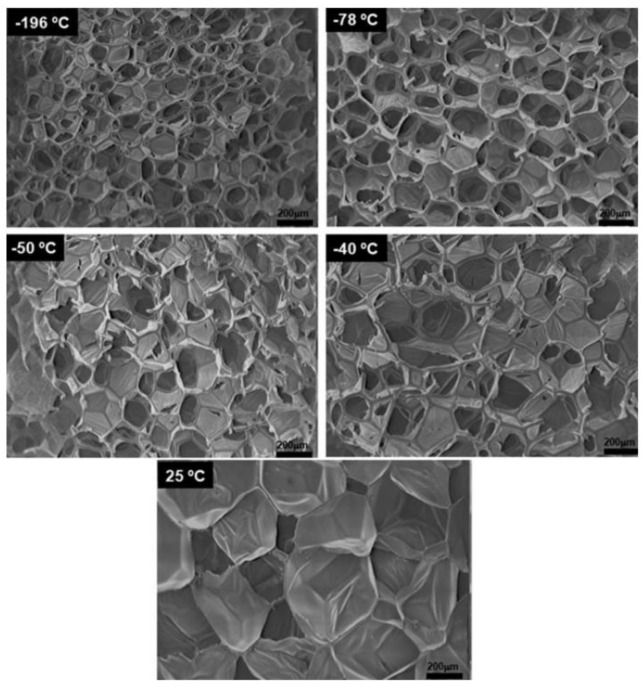
SEM images showing pore formation for PLLA scaffold during solvent extraction at different temperatures. Reproduced from [120].

Mallick et al. [120] fabricated highly porous and interconnected 3-D network PLLA/BG 45S5 scaffolds by freeze-drying technique. As shown in Figure 5, pore size and porosity can be controlled via freezing temperature employed. Santos et al. [121] fabricated PCL/BG 58S porous composite and characterized the mechanical and cell viability properties. Maquet et al. [122] prepared two series of porous composites based on PLGA/BG and PDLLA/BG at three different contents by freeze-drying method. They studied the microstructural, bioactivity and mechanical properties. The degradation rate was adjusted by varying the nature of the polymer and the content of BG in the matrix. The studies of Rezabeigi et al. [123,124] shown a high porosity and interconnected PLA/BG scaffolds with a combination of different size of pores. Conoscenti et al. [125] compared the bioactivity, microstructural and mechanical properties during in-vitro conditions of resorbable scaffolds based on PLLA and 45S5 or 13-93 BG. The PLLA/13-93 BG composites presented a better particle dispersion into the matrix and more homogeneous pore size.

In the study of Dziadek et al. [61], PCL/BG composite with two different compositions of BG were studied following three different preparation methods: solvent casting particulate leaching (SCPL), solid–liquid phase separation (SLPS) and phase inversion (PI). In all of the methods, PCL was firstly dissolved. The different techniques presented similar bioactive properties. However, each technique presented a different porosity and pore size. In their in-vitro studies they demonstrate the low cytotoxicity (mostly due to the production methods) and the bioactive behavior of PCL/BG composites scaffolds.

The different investigations presented above demonstrate the bone growth promotion of scaffolds. However, owing to the low mechanical properties, polymer-based scaffolds can be used only for non-load-bearing applications.

Polymer solution techniques present significant problems to moving from laboratory to industrial scale as the long-time fabrication, the presence of toxic chemical substance in the final product, and the low mechanical properties. Moreover, even if poly(α-hydroxy acids) solvents like chloroform or acetone evaporates rapidly in room conditions, they can remain during several months in atmosphere before been completely degraded or easily dissolved in water [126,127].

**Table 1:** Principal properties of solvent route molding investigations. With * the compression, ** tensile and *** flexural modulus/strength (not filled cells mean no data).

Composite	Modulus (MPa)	Strength (MPa)	Strain at Break (%)	Porosity	Pore Size (µm)	Particle Size (µm)	Content (wt%)	Technique	By
Cancellous bone	20–50 **	2–12 */7.4 **							[127]
Cortical bone	3000–30000 **	130–180 */60–160 **							
PLA-PEG	2.5 **	10.1 **					0	Solvent Casting	[114]
PLA-PEG/45S5	4.9 **	18.5 **				<38	1		
PDLLA		18 **					0	Solvent Casting	[115]
PDLLA/45S5		12.3 **				20	15		
PDLLA/58S		50.6 **				1	15		
PDLLA/m58S		29.3 **				1	15		
PCL	0.13 **							Solvent casting	[71]
PCL/45S5	0.43 **					6	5		
PCL/45S5	0.82 **					0.25	5		
PCL/45S5	0.46 **					<0.1	5		
PCL	800 **							Solvent casting	[116]
PCL/SrBG	5600 **					0.4	10		
PCL/CaBG	5500 **					0.2	10		
PCL/PLGA	1500 **	31.5 **						Solvent casting	[109]
PCL/PLGA/BG	3400 **	38 **				<5	20 vol%		
PCL	280 **	148 **	240	-	-	-	-	Solvent Casting	[117]
PCL/45S5	190 **	51 **	2.4	-	-	3.7	50		
PCL/13-93B3	146 **	41 **	27	-	-	4.0	50		
PCL/45S5/	190 **	44 **	5.5	-	-	3.7–4.0	50		
13-93B3									
PLGA	14 **	2.1 **	460			155		Solvent casting	[110]
PLGA/BG	10 **	1.9 **	390			175	20		
PLGA/BG	10 **	2 **	350			170	40		
PLGA		4.1 *–1.9 ***		80				Gas foaming	[119]
PLGA/g-BG		5.5 *–2.8 ***		80		0.04	20		
PLGA	7.6 *	1.4 *		73–85	120–320		0	Solvent/gas foaming	[118]
PLGA/BG	13 *	1.8 *					10		
PLGA/BG	18 *	2.1*					20		
PCL	0.08–0.19 *							Freeze-drying	[74]
PCL/45S5	0.13–0.23 *			90	50–300	<45	25		
PCL/45S5	0.16–0.25 *			90	50–300	<46	50		
PLLA/45S5 BG	-	0.8–0.3 *	-	81–91	250–1100	<2	-	Freeze-drying	[120]
PCL/58S BG	46 *	4.5*		72		18	10 vol%	Freeze-drying	[121]
PDLLA	13.6 *			90	10–100			Freeze-drying	[122]
PDLLA/45S5	21 *			90	10–100	25	50		
PLGA	9.8 *			90	10–100				
PLGA/45S5	26.5 *			90	10–100	25	50		
PLA/45S5				91.4	110	1.82	2	Freeze-drying	[123]
PLA/45S5				89.3	72	1.82	2		
PLA/45S5				87.9	46	1.82	2		
PLA/45S5				85.3	40	1.82	2		
PLLA	6.3 **				60			Freeze-drying	[125]
PLLA/45S5	6.5 **			88.5	25	11.26	5		
PLLA/13-93	8.2 **			88.5	60	8.86	5		
PCL/BG				57	10–100	<50	21 vol%	SLPS	[61]
PCL/BG				65	10–100	<50	21 vol%	Freeze-drying	
PCL/BG				90	>100	<50	21 vol%	SCPL	

### 4.2. Thermal Route

The results of different studies presented in this section are summarized in Table 2.

#### 4.2.1. Injection Molding

The injection molding fabrication of poly(α-hydroxy acids)/BG composites is divided in two steps. In a first time, poly(α-hydroxy acids) and BG particles are mixed following a thermal extrusion [62,69] or a solvent casting [59] process to obtain composite pellets with well-dispersed particles. Afterwards, composites are processed into different shapes at high temperature and under pressure by injection molding. Generally, compared to other manufacturing process, injection molding parts present better mechanical properties thanks to the lack of porosity.

**Figure 6:**
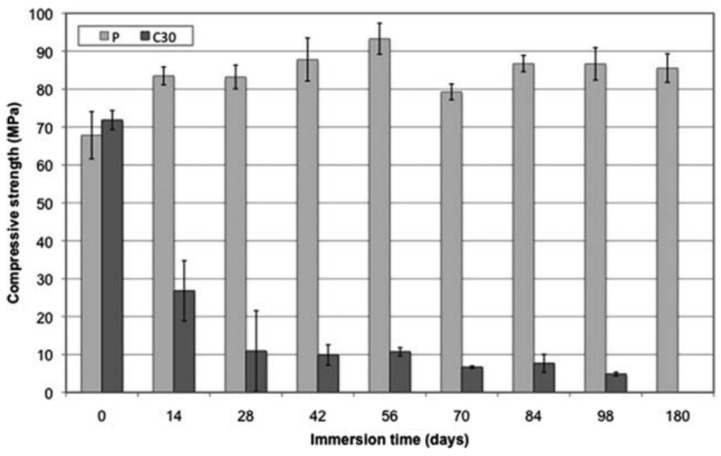
Compressive strength of the polymer P (light gray) and the composite C30 (dark gray) in function of immersion time in PBS. Reproduced from [59].

Ji et al. [62] investigated the mechanical properties and bioactivity of composite based on PCL filled with nanoparticles of BG. Although the tensile strength remained almost the same, the addition of BG particles, increased in elastic modulus. PCL/nBG composites exhibited an excellent bioactivity after being immersed in SBF (Simulated Body Fluid) fluid. However, they showed a faster degradation behavior. Simpson et al. [69] presented a detailed study of PLGA with different bioactive fillers of the thermal and mechanical properties. Compared to HA, composites filled with bioactive particles exhibited lower mechanical properties due to the premature degradation of the PLGA matrix and poor particle/matrix adhesion. Vergnol et al. [59] used a two-step process to prepare PDLLA/BG composites. The composites pellets were fabricated by solvent technique and afterwards, they were molded by injection. As observed in Figure 6, the in-vitro tests revealed that composite systems presented a faster degradation rate. In addition, poly(ethylene-vinyl alcohol)/BG composites exhibited the same behavior [128]. In order to reduce the polymer degradation at high temperature, Lacambra et al. [66] fabricated a PDLLA/BG composite by a coupled thermal extrusion with direct injection molding process. 

These different studies observed that the bioactive particles in the resorbable polyester matrix led to a significant decrease in the strain at break and tensile strength. Furthermore, they observed only an elastic deformation without yield stress and strain softening during the tensile experiment for the composites, suggesting a brittle fracture.

#### 4.2.2. Hot-Pressing 

Hot-pressing molding consists on heating a polymer (or composite) in a closed mold, under a controlled temperature and pressure, to take the shape of the mold cavity. The team of Mehboob et al. modelled the mechanical behavior of a FGM bone plate based on a multilayer FGM PLA/BG composite [129]. Afterwards, they studied the in-vitro mechanical properties evolution of the same FGM composite during the immersion in a phosphate-buffers saline (PBS) solution [130]. The FGM were fabricated by hot-pressing molding. They investigated the healing of critical segmental bone fractures by following the in-vitro formation on new tissues. A recent study fabricated scaffolds from PCL/BG microspheres by a low-temperature process [131]. In this case, the porosity was ensured by the partially sintering of microspheres surfaces. 

**Table 2:** Principal properties of extrusion-injection molding investigations. With * the compression, ** tensile (not filled cells mean no data).

Composite	Modulus (MPa)	Strength (MPa)	Strain at Break (%)	Porosity	Pore Size (µm)	Particle size (µm)	Content (wt%)	Technique	By
Cancellous bone	20–50 **	2–12 */7.4 **							[127]
Cortical bone	3000–30,000 **	130–180 */60–160 **							
PCL		40 **						Extrusion-injection molding	[62]
PCL/nBG		20 **				0.05–0.09	10		
PCL/nBG		20 **				0.05–0.09	20		
PCL/nBG		20 **				0.05–0.09	30		
PCL/nBG		17.5 **				0.05–0.09	40		
PDLLA	2100 *	82 *						Extrusion-injection molding	[66]
PDLLA/45S5 BG	2200 *	78 *				40–500	10		
PDLLA/45S5 BG	2500 *	75 *				40–500	30		
PDLLA/45S5 BG	2200 *	64 *				40–500	50		
PLGA/45S5 BG	3500 *	69 *				35.3	25 vol%	Extrusion-injection molding	[69]
PLGA/ICIE4 BG	5900 */7500 **	93.1 */35.8 **				5.2	25 vol%		
PLGA/HA	5900 */8800 **	93.1 */51.7 **				3.8	25 vol%		
PDLLA		68 *					0	Extrusion-injection molding	[59]
PDLLA/45S5 BG		72 *				3.5	30		
PVA	1860 **	42.3 **	14.7				0	Extrusion-injection molding	[128]
PVA/45S5 BG	3540 **	50.7 **	2.5			38–53	10		
PVA/45S5 BG	3770 **	38.6 **	1.8			38–53	40		
PCL	5 *			85.9	183			Hot-pressing and Salt-leaching	[60]
PCL/HA	8.2 *			86.2	180	0.02	20		
PCL/45S5	8.6 *			87.5	177	66.4	20		
PCL	34 **	2.2 **		44.5	100		0	Microsphere sintering by hot-pressing	[131]
PCL/BG	47 **	2.7 **		44.5	100		5		
	37 **	2.1 **					10		
	30 **	1.9 **					20		
PDLLA	2.2 *	0.42 *		92	920			Gas foaming by SSF	[132]
PDLLA/BG	4.9 *	0.7 *		91	270	50	10		
PDLLA/BG	7.3 *	1.2 *		79	190	50	30		

#### 4.2.3. Solid-State Foaming (SSF)

Mohammadi et al. [132] fabricated a PDLLA/BG composite foam via a solid-state foaming (SSF) using CO_2_. Composite pellets were fabricated by melt-extrusion under a flow of nitrogen to reduce the polymer degradation. Afterwards, the different specimens were processed by hot-pressing molding. The dense parts were saturated with CO_2_ under pressure and foaming was conducted at 80 °C. Compared to the other foaming techniques, SSF presents the advantage of being a solvent free process. 

#### 4.2.4. Salt-Leaching

**Figure 7:**
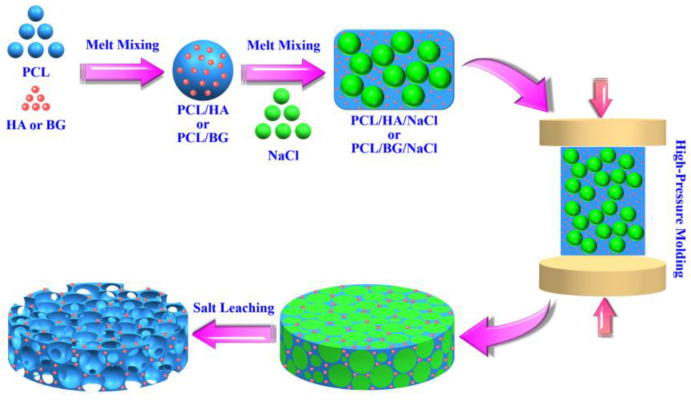
Schematic diagram of fabricating porous PCL composite scaffolds. Reproduced from [60].

Scaffolds can also be fabricated by a salt-leaching technique. In this case, salts powders are used as pore generation. This technique can be coupled with a solvent or melt method such as hot-pressing molding, solvent casting or robocasting [60,133,134]. At the end, the systems are immersed in water to dissolve the salts and create the composite scaffolds. Figure 7 exhibits the different manufacturing steps of scaffold fabrication by hot-pressing molding combined with salt leaching. Yin et al. [60] studied the bioactivity and mechanical properties of PCL/HA and PCL/BG scaffolds. The composites were firstly mixed by extrusion compounding with NaCl salts and then molded at high pressure. 

### 4.3. Additive Manufacturing (AM)

Additive manufacturing (AM) techniques have attracted attention in bone tissue engineering during this lasts years [8]. AM techniques are considered the group of fabrication process that manufacture parts by a gradually addition of materials. These techniques are advantageous than traditional methods since it can customize, repeat architectures, fabricate complex designs, are low cost, and are highly efficient [12]. One of the key advantages of AM is the ability to produce tailored devices adjusted to each patient. However, these technologies are material-based dependent, since there are some mechanisms that works only for some specific materials. Thus, different publications have shown the possibilities of polymer, ceramic and metal-based AM techniques for bone regeneration applications [6,8,135].

Although a huge number of polymer-based publications using different AM techniques can be find in the literature, the main objective of this review is to present mainly the investigations using poly(α-ester)/BG composites. Table 3 shows the advantages and limitation of each AM technique. At the end of each section we present a table summarizing the principal mechanical and microstructural properties of the systems studied in the literature.

#### 4.3.1. Fused Deposition Modelling (FDM)

Fused deposition modelling (FDM) is the most commonly-used AM technique. The extruded is melted through a heated nozzle and deposited layer-by-layer (with an accuracy on the order of 100 µm) to create a 3D part [142]. The final properties like anisotropic mechanical properties or surface quality are determined by nozzle dimensions and polymer viscoelasticity. 

Korpela et al. [143] demonstrate the printability of a PCL/BG biocomposites. Firstly, the composite printability was more challenging to print than PCL: the adhesion between adjacent layers was weaker and the extrusion flow was unstable due to the high viscosity. Afterwards, the printing parameters like the nozzle temperature, porosity or layer orientation were optimized. The composites presented a similar mechanical behavior to PCL scaffolds. 

For the purpose of show the interest of BG particles in biocomposites for bone tissue regeneration, Alksne et al. [144] compared the in-vitro results between scaffolds of PLA/HA and PLA/BG manufactured by FDM. A powder mix of PDLLA/HA and PDLLA/BG at 10 wt% content was extruded at 140–145 °C to create a filament with a diameter of 1.28–1.6 mm. Different in-vitro tests have been realized (DPSC, cell adhesion, DPSCs migration and proliferation and osteogenic differentiation) to compare the osteoconductive, osteoinductive, and biocompatible properties. The results confirmed that the bioactivity properties of PDLLA/BG were better than PDLLA/HA. 

Distler et al. [145] fabricated and studied the properties of PLA/45S5 BG filaments for 3D scaffolds manufacturing by FDM. The µCT images confirmed the interconnected porosity of scaffolds and BG particle distribution into the PLA matrix. The different composite filaments presented an ultimate tensile strength between 35 and 60 MPa.

**Figure 8:**
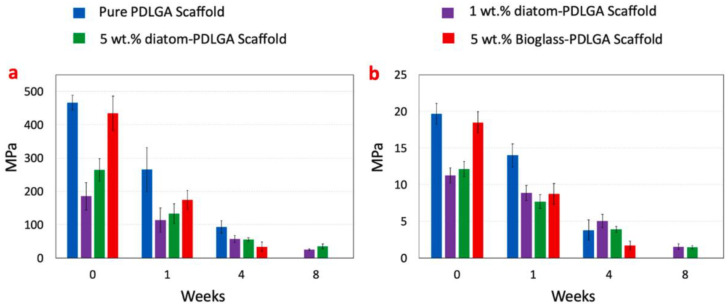
Results from compression test of immersed samples in PBS (**a**) Average Young’s modulus, (**b**) Average yield stress. Reproduced from [146].

The mechanical properties of PDLGA/45S5-BG 3D scaffold fabricated by FDM during in-vitro degradation are significantly affected by the presence of BG. In the in-vitro study of Han et al. [146], the composites filled with different BG presented a decrease of the mechanical properties from the first week of immersion. A structural decomposition appeared up to 4 weeks of immersion for scaffolds filled with non-thermal treated BG. As shown in Figure 8, even if scaffolds with thermal treated particles presented lower mechanical properties after fabrication, they presented higher mechanical properties up to 4 weeks of immersion and maintained their shapes and porous structures during the first 8 weeks. The results of different studies presented in this section are summarized in Table 4.

#### 4.3.2. Robocasting or Direct Ink Writing

**Figure 9:**
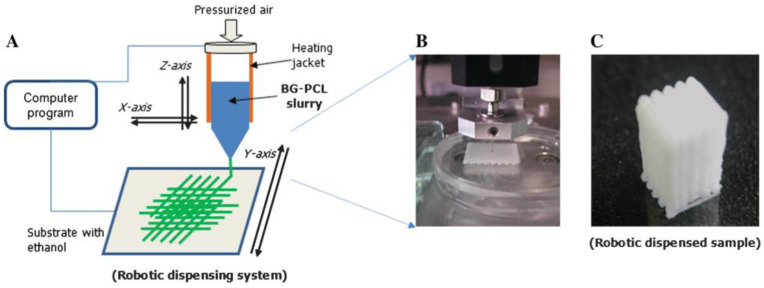
(**A**) Schematic diagram of robotic dispending method, (**B**) enlargement of the dispensing nozzle and deposited fiber mesh, and (**C**) three-dimensional PCL/BG composite scaffold. Reproduced from [147].

Robocasting or Direct ink writing is a type of 3D material extrusion-based technique. Briefly, this technique consists to extrude a polymer solution or a melted polymer through a nozzle using a force-controlled plunger like a screw, a piston or air pressure to control the mass flow rate. Figure 9 shows a schematic diagram of robocasting process.

To understand and validate the robocasting process to fabricate PCL/BG scaffolds for bone tissue engineering, Oh et al. [147] studied the morphological changes (particle dispersion, pore size and filament diameter) during immersion in SBF. The composite paste was prepared by mixing PCL and BG with acetone at 50 °C. The rheological properties of the mixture were examined in order to ensure the printability and the final geometry of the scaffold structure. 

Yun et al. [134] fabricated a PCL/BG scaffold with three pore dimensions via a combination of robocasting, salt leaching and mesoporous BG. During the scaffold fabrication the NaCl had an also an effect of stiffening supporter avoiding the collapse of the structure. In order to analyze the effect of porosity in bone tissue regeneration devices, three compositions with different porosity and pore dimension have been studied. The mechanical strength was inversely proportional to porosity. Scaffolds presenting high macro-porosity exhibited an increase of the loss modulus (E″). Their sponge-like plastic nature and bioactivity make them interesting for minimum invasive surgery and articular cartilages reconstruction.

Several studies enhanced the biomedical response by introducing metallic ions or drugs. The in-vivo studies of Gomez-Cerezo et al. [89] carried out into cavity defects, proved excellent bone regeneration properties for scaffolds of PCL/mBG with an antiosteoporotic drug fabricated by robocasting. Sánchez-Salcedo et al. [83] studied the in-vitro antibacterial of PCL/BG scaffolds with ZnO to decrease in infection rates during implantation. Zhang et al. [82] fabricated a PVA/mBG scaffold with Sr ions to reduce inflammatory response as soon after the device implantation. Zhang et al. [90] introduced magnetite (Fe_3_O_4_) nanoparticles into PCL/BG composite for cancer treatment. Wang et al. [97] fabricated an SME scaffold using a Pickering emulsion of poly(D,L-lactide- co-trimethylene carbonate) (PLMC)/BG composite.

Murphy et al. [133] studied the cell viability and proliferation properties of scaffolds fabricated with two inks: adipose stem cells (ASCs) and PCL/13-93B3/chloroform mix. The formation of a porous filament by the chloroform evaporation increased the glass dissolution, bioactivity and polymer bulk degradation. Kolan et al. [148] studied the mechanical properties and biological response of bi-material scaffolds based on PDLLA/BG 13-93B3 and Bioink (Alginate + Gelatin + ASCs).

In order to improve the regeneration of osteochondral defects, Barbeck et al. [149] fabricated a bi-layered PLA and PLA/BG scaffold. The printing conditions and paste composition used in this study have been previously optimized by Serra et al. [150]. They analyzed the in-vitro degradation in SBG of scaffolds by following the mass loss, the weight average molecular weight (Mw) loss and the compressive modulus. Even if the studied properties decreased faster in PLA/BG than PLA, PLA/BG scaffolds kept their structural integrity during the in-vitro experimentation (eight weeks). For in-vivo study, the addition of BG showed a bioactive response of scaffolds.

Baier et al. [151] prepared a 3D PCL/45S5 BG composite scaffolds by direct ink writing at high temperature. The raw composite pellets were fabricated by solvent casting. The rheological investigation confirmed the printability of PCL/BG composites at different BG content and shear rates. 

Some studies used a paste based on Pluronic F-127 mixed with BG to create 3D scaffolds. For example, Nommeots-Nomm et al. [152] observed the effect on ink printability as a function of BG nature. In some cases, a debinding and sintering step were used to create highly porous BG scaffolds. Barberi et al. [153] and Baino et al. [154] studied the compressive behavior and bioactivity of BG 47.5B scaffolds in SBF. The formation of cHA on the surface of BG 47.5B scaffolds after immersion in SBF and the ion concentration profile confirmed the bioactivity.

The team of Eqtesadi et al. [155,156,157,158] fabricated scaffolds of 45S5 BG with an optimized paste composed of caboxymethyl cellulose CMC (0–2 wt%), a polyelectrolyte dispersing agent, deionized water and 45vol% of BG. After the debinding and sintering step (at 500 and 1000 °C respectively), the mechanical properties of scaffolds were tested. Their recent studies [159,160] shown the improvements on compression and flexural properties after a PCL and/or PLA infiltration by immersion in a polymer melt bath at 227 °C. As previously notice [23,65,66], the authors observed a chemical degradation of PLA in contact with BG at high temperature. Furthermore, the increase of crystallinity degree of BG by increasing the sintering temperature reduced the bioactivity kinetics under in-vitro conditions [66]. Finally, Motealleh et al. [73] studied the mechanical properties and in-vitro degradation of BG scaffolds coated with different synthetic polymers (PCL, PLA) and natural polymers (alginate, chitosan, gelatin). Scaffolds with a polymer coating exhibit a higher compressive strength, a strain energy density and weight loss than BG scaffolds. Although the coating by the solvent route shown a better infiltration and a non-degradation of resorbable polyester, the mechanical properties of scaffolds with a polymer coating were higher than those of non-coated scaffolds, whereas the coating technique. The results of different studies presented in this section are summarized in Table 5

#### 4.3.3. Electrospinning

Electrospinning is one of the approaches used to manufacture micro-fibrous scaffolds based on medical degradable polymers filled with bioactive ceramics. Electrospinning is a processes by which a steady stream of an electrically charged polymer material (in a dissolved or melted state) is drawn into microfibers under the action of electrostatic forces [139]. The high voltage electric field between the nozzle and the collector plate (generally between 4 and 30 kV) is the principal responsible for drawing down the original diameter of the material. However, other electrospinning process parameters such as solvent, polymer concentration, flow rate or temperature can also influence the microfibers diameters and final mechanical properties [160].

**Figure 10:**
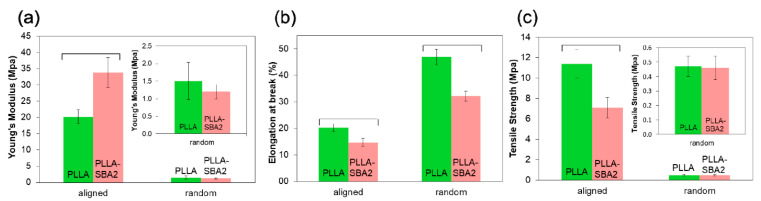
Young’s modulus (**a**), elongation at break (**b**) and tensile strength (**c**) of random and aligned fibers. Results are expressed as (mean ± standard deviation). Bars show statistically significant differences (*p* < 0.05). In the inset of (**a**,**c**) a zoom view of the properties of randomly oriented fibers is reported. Reproduced from [161].

Kouhi et al. [160] studied the electrospinning fabrication of a PCL/ chloroform/methanol solution with different contents of BG particles. The electrospun nanofibrous presented a good tensile strength and bioactivity. Konyalı & Deliormanlı [71] fabricated a PCL/13-93BG composite by electrospun method using acetone in solution. They studied the effect of BG morphology on in-vitro bioactivity of composite scaffolds. Serio et al. [161] fabricated two types of fiber mats of PLLA/BG by electrospinning: aligned and random. As observed in Figure 10, compared to neat polymer fibers, PLLA/BG composites shown an increase of the elastic modulus. Moreover, composite fibers shown a ductile behavior. A cell culture of ST-2 confirmed that PLLA/BG fibers promote an effective and viable environment for cellular colonization.

Liverani et al. [96] studied the feasibility of PCL-TES (triethoxysilane-terminated)/BG shape memory effect (SME) manufactured by electrospinning. The samples presented an excellent shape fixity and shape recovery. 

Moura et al. [89] studied the mechanical strength and bioactive response of a PCL/nBG and PCL/nBG doped with metallic ions (silver nanoparticles and cobalt ions) mats fabricated by electrospinning process. These mats presented a tensile strength (14–27 MPa) and an elongation (103–167%) close to the values of human skin (5–30 MPa and 35–115% respectively) and makes them interesting to be used as a skin regeneration membrane. Canales et al. [162] studied the mechanical properties and cell viability under in-vitro conditions of PLLA/BG scaffolds with MgO fabricated by electrospinning. The results of different studies presented in this section are summarized in Table 6.

#### 4.3.4. Melt Electrospinning Writing (MEW)

**Figure 11:**
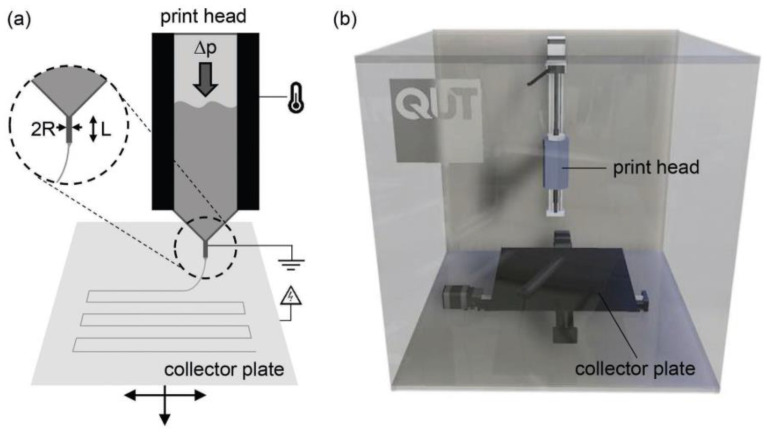

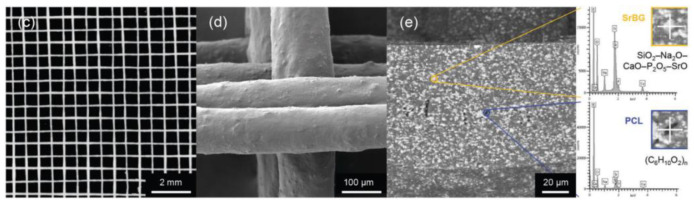
(**a**) Schematic diagram of the MEW process using an applied pressure (ΔP) to extrude a molten polymer or polymer solution through a nozzle with a radius R and a length L to a moving collector plate. (**b**) Visualization of the MEW device. (**c**–**e**) SEM image of microfiber morphology. Reproduced from [165].

Melt electrospinning writing (MEW) is a high-resolution additive manufacturing technique that facilities the fabrication of scaffolds with polymer microfibers. This additive manufacturing technique is a combination of electrospinning and robocasting process [140]. As electrospinning, the scaffolds can be fabricated by solvent or thermal route. In the case of thermal route, the composite pellets are loaded into a syringe and melted and for solvent route, the polymer solution is directly extruded through the syringe by air pressure or pneumatic system [163,164]. A monitored moving collector plate and a high potential difference between the nozzle and plate leads to the layer-by-layer fabrication of micro fibrous scaffolds scaffolds (Figure 11).

Hochleitner et al. [166] proved the processability of PLA/PEG with 5 wt% of 45S5 BG scaffold using the MEW. Paxton et al. [165] studied the rheological behavior of PCL/SrBG (strontium bioactive glass) scaffold fabricated by MEW method. The PCL/SrBG composite was prepared by a micronization of SrBG particles and mixing with a PCL/chloroform solution. In this study, they used the Ostwald model and a non-Newtonian shear rate model to predict the composite printability according to process parameters such as printing pressure, temperature, Sr BG content, average extrusion velocity, and the radius, R, and length, L, of the cylindrical nozzle. 

#### 4.3.5. Selective Laser Sintering (SLS)

The SLS is a layer-by-layer process where a laser energy source is employed to raise the temperature and fuse the powder material particles together without completely melting the material. The principal parameters to control are: the laser power, the sintering speed, the spot diameter at focus, the powder temperature, the layer thickness, the powder polymer, and filler size (Figure 11). The final parts present interconnected microporous due to the non-completely fusion of particles which can facilities the cell attachment and fluids transport through them. The parts fabricated by SLS offers the advantage of having good mechanical properties, good resolution and a no-need of support structures, post-processing step, or solvents.

**Figure 12:**
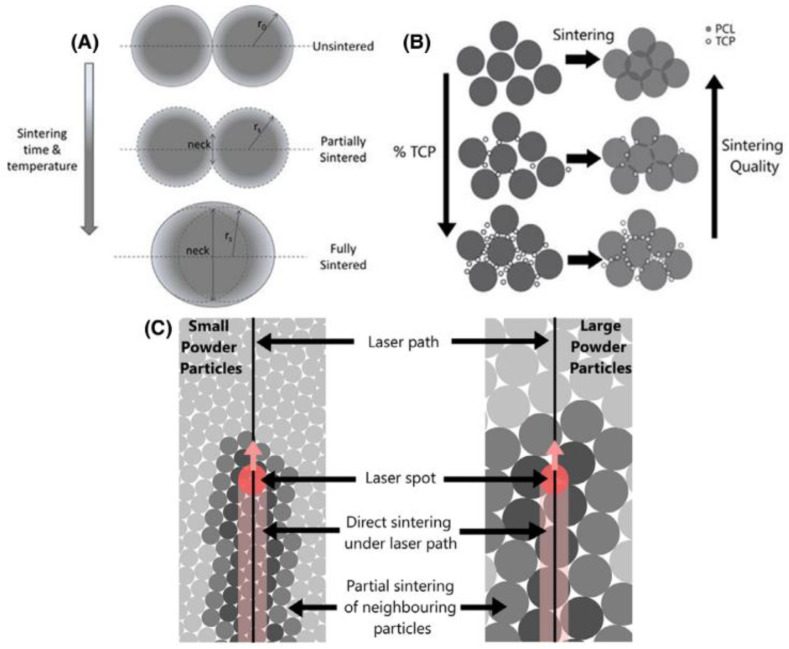
(**A**) Healing of individual polymer particles to form partially sintered and fully sintered material as a function of time and temperature. (**B**) Illustration of the effect of increasing filler content on the sintering of composite materials. An increase of filler content difficult the polymer sintering. (**C**) Schematic of sintering of powder particles with different particle sizes. Regions directly under the laser path are fully sintered, and heat is transferred to surrounding particles through contact points to form partially sintered regions. Higher partial sintering and lower porosity occurs for small particle sizes. Reproduced from [168].

The fabrication of complex geometries with a controlled micro- and macro-pore size and good mechanical properties needs the control and optimization of the sintered level, the effect of the polymer and filler particles size or the layer thickness (Figure 12). For example, Do-Vale-Pereira et al. [167] optimized the PDLLA/58S-BG scaffolds fabrication by modifying the process parameters such as the laser energy density and the BG content (0, 10, 20 and 30 wt% of BG). 

**Figure 13:**
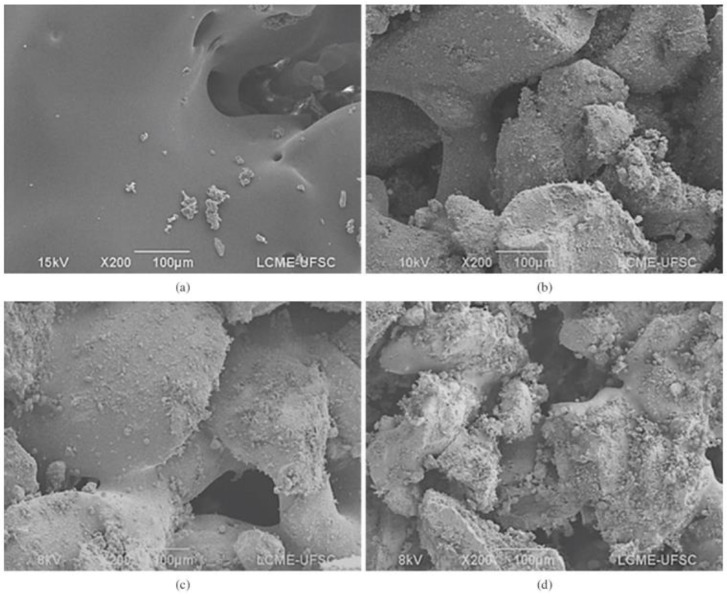
Micrographs with 200× of magnification. (**a**) pure polymer; (**b**) polymer with 10 wt% of BG; (**c**) polymer with 20 wt% of BG; (**d**) polymer with 30 wt% of BG. Reproduced from [167].

Figure 13, from the study of Doyle et al. [168], shows the heling process of polymer particles, the effect of particle size and the effect of filler content during the SLS fabrication. Salmoria et al. [169] studied the properties of Poly(L-co-D,L)lactic acid (PLDLA)/58S-BG scaffolds fabricated by SLS. They characterized the microstructural, flexural, thermomechanical and cell viability properties at different BG contents.

Karl et al. [170] fabricated a PLGA/BG composite microsphere for SLS. This powder was produced by a solid-in-oil-in-water (s/o/w) emulsion method. They studied the influence of the process to produce porous microparticles, the influence of BG content on the macro/microstructure and the SLS application to fabricate structures with these microspheres. These kind of microporous particles could have others biomedical applications such as carriers for drugs, absorption of substances, pulmonary drug delivery and tissue regeneration [5,171]. 

Xu et al. [172] studied the effect of polydopamine as a cross-linking bridge between mesoporous BG and PLLA to improve the interfacial interaction during the scaffold fabrication. Afterwards, Xu et al. [173] studied the mechanical and biological properties of a scaffold based on a blend of PLA and PGA (PGPL) filled with polydopamine functionalized mesoporous BG with dexamethasone (DEX). The mBG had promoted cell proliferation and DEX increased the alkaline phosphate (ALP) activity of osteoblasts and thus bone formation and calcification. 

Qian et al. [174] introduced Ag ions into mesoporous BG nanoparticles since it presents an antibacterial capacity. The mechanical tests as well as the in-vitro test confirmed the good mechanical properties, admirable antibacterial ability and cytocompatibility. 

Some studies, proposed a debinding and sintering step to eliminate the organic phase and obtain BG scaffolds with high porosity. In this case, the authors used binders like stearic acid (SA) as a supporting material: during the SLS process the laser melts the stearic acid which bonds the BG particles. Afterwards, the scaffolds are post-processed at 550 °C to burn out the binder and to sinter the 13–93 glass particles. This thermal treatment did not affect the amorphous nature of the BG, which could otherwise have an impact on the bioactivity [31,66]. The works of Kolan et al. [175,176,177] studied the morphological, mechanical and bioactive properties of BG13-93 scaffolds elaborated by SLS technique. They optimized the effect of pore size, porosity, blinder (SA) content and layer thickness. For example, scaffolds with high pore size and porosity do not have enough strength to remove the unsintered powder from the pores. The optimization of the heating rate for the debinding and sintering step enabled to control the densification and therefore reduce the internal voids. Furthermore, decreasing the layer thickness allowed to reduce the stearic acid content, delete the delamination between layers, obtain higher strength and a better surface finish. The scaffolds presented the appropriate morphological and mechanical properties for non-load-bearing applications since they had a highly interconnected porous network, a suitable pore size (300–800 µm) to facilitate the fluid body circulation and the compressive strengths obtained was significantly higher than that of trabecular bone. The results of different studies presented in this section are summarized in Table 7.

#### 4.3.6. Stereolithography (SLA)

The stereolithography (SLA) is based on photo-linking of a liquid polymer resin into a 3D architecture. This AM technique is a layer-by-layer process where a UV or laser light photopolymerizes the surface corresponding to the 3D part. For this technique, two approaches have been observed in the literature (Table 8): utilization of a resorbable photopolymer as a matrix for the final bioactive composite [178] or utilization of a photopolymer as a sacrificial agent [178,179,180,181,182,183].

**Figure 14:**
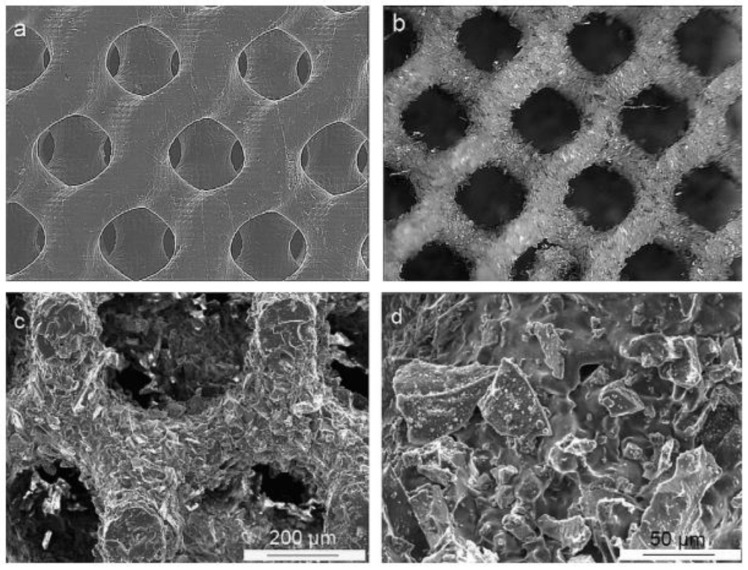
(**a**) SEM image of a BG scaffold, (**b**) optical stereomicroscope image of a PCL scaffold with 20 wt% of BG and (**c**,**d**) SEM images of a surface of a PCL/BG scaffold. Reproduced from [178].

Figure 14 exhibits the scaffolds of PCL/BG with a well-defined architecture (gyroid pores) fabricated by Elomaa et al. [178] using the SLA process. In their study, PCL monomer was mixed with a photoinitiator and a solvent dye at different BG contents. The processing parameters chosen were: 12 s of exposure time, 1600 mW/dm^−2^ of light intensity and a layer thickness of 50 µm. Finally, the non-photopolymerized PCL was removed by immersion in a solvent mixture of acetone and isopranol. The scaffolds containing 20 wt% shown a compressive strength between 2.5 and 3.4 MPa (for dry and wet conditions, respectively) and good bioactive properties during in-vitro test. They observed that the compressive strength and bioactivity was improved by increasing the BG content.

Tesavibul et al. [182] shown for the first time the 45S5 BG scaffold fabrication with 3D interconnected pores by lithography-based AM process. After the green part fabrication, the solvent and the polymeric blinder were eliminated and the sample sintered. Thavornyutikarn et al. [179] fabricated different scaffold architectures to show the structure with the most controllable properties such as the compressive strength, the pore interconnection and shrinkage. In this investigation, different pore shapes and pore sizes with the same level of porosity were studied. The combination of an optimum scaffold structure and a thermal pre-treatment of BG powders exhibited mechanical properties close to those of cancellous bone. They used an acrylate-based photopolymer resin as a sacrificial agent mixed with BG (41 vol%). After the 3D printing, the scaffolds were heated at 550 °C during 3 h to burn out the resin. Ma et al. [181] optimized the debriding and sintering parameters of 45S5-BG scaffolds fabricated by SLA with a photosensitive resin. The optimized parameters were a sintering temperature at 1000 °C, heating rate of 5 °C/min and a holding time of 0h. Gmeiner et al. [183] increased up to 124 MPa the bending strength of dense 3D printed BG fabricated by SLA process. The slurries presented a high content (70 wt%) of well-dispersed BG particles. 

The FT-IR technique was used by Kang et al. [180] to verify the completely non-presence of blinders after the sintering step. They fabricated scaffolds of 45S5 bioactive glass with photocurable and acrylate blinders. The objective was to obtain dense structures from polymeric suspension with high BG particles loading. However, a high concentration of BG particles increased the viscosity of the suspension, hindering the printability. Analyzing the rheological behavior of the suspensions, the authors calculated the printable composition with the highest BG content. The scaffolds elaborated with the highest BG content (60% of blinder and 40% of BG) and with the highest viscosity, show the best mechanical and morphological properties: less shrinkage, a high relative density and high biaxial flexural strength. 

## 5. Conclusions

The level of bone injury, the place of bone fracture or the age of the patient plays an important role in the choice of the material and manufacturing process. However, there exists a large list of biocompatible materials for bone tissue engineering. Moreover, the bone implant fabrication involves several processes such as structural design, manufacturing technique and additional treatments. This review presents the different manufacturing techniques with poly(α-hydroxy ester)/BG composites and classifies the recent papers as a function of the processing method.

The different studies discussed in this review allow us to conclude that poly(α-hydroxy ester)/BG composites are a promising resorbable material for the production of scaffolds and dense medical devices that may exhibit a controllable porosity, mechanical properties, degradation rate, in-vivo, and in-vitro bioactivity.

In the field of resorbable bioactive composites recent studies focus on scaffold fabrication since they show a good osteogenesis and permit the body fluids circulation. However, the use of traditional approaches for scaffold fabrication (e.g., solvent casting, particulate leaching, phase separation, electrospinning or freeze drying) presents a limited capacity to ensure sufficient mechanical properties and control the internal structure like porosity, pore size, pore morphology and pore interconnectivity. Additive manufacturing techniques can provide scaffolds with a controlled porosity, pore size, pore geometry and ensure a 3D pore interconnectivity and better mechanical properties. Unfortunately, the mechanical properties of these scaffolds are significantly lower than bone and cannot be used for load-bearing applications.

Shape memory materials (SME), functionally graded materials (FGM) or materials with therapeutic applications are, and will be the focus of bone tissue engineering investigations. These new materials present several advantages as the reduction of the incision length, mimic the bone porosity, control and increase the strength, or includes an antibacterial/anti-inflammatory effect. 

Although SLS, SLA or MEW have demonstrated their ability to create 3D geometries with interesting biomedical properties, FDM and robocasting are the most frequently used AM techniques to fabricate resorbable composites scaffolds and dense parts. Interestingly, 3D material extrusion techniques can combine multiple extrusion systems to fabricate multi-material parts. Hence, the differents 3D extrusion process are a promising feasible alternative to fabricate low-cost and modulable FGM either with a structural or compositional gradient. 

In most of the studies, it appears that the study of the melted or solid rheological behavior of composites is not very developed while it could be very helpful to optimize manufacturing processes and consequently the resulting properties. 

Besides, further investigations are needed regarding the mechanical properties evolution of poly(α-hydroxy ester)/BG composites fabricated by 3D material extrusion techniques under in-vitro degradation conditions to better understand the relationship between the composites resorption and their mechanical properties change.

## 6. Towards Resorbable Load-Bearing Applications

The works presented in this review provides an overview of the clinical needs and the main manufacturing techniques for composites based on resorbable polyester matrix filled with bioactive glass. The combination of a bioactive glass and a polyester provides biocomposites with attractive properties when used as a medical osteosynthesis device. The bioactive glass ensures a bioactive material while the polyester can bring an easily processability and mechanical properties that are essential for load-bearing applications.

The final properties of composites are influenced by the manufacturing techniques, but also by the size, shape and composition of the bioglass. The bioactive glass 45S5 is a well-known biomaterial for the bone regeneration applications since it presents a high bioactivity index [184]. However, polyester matrix filled with bioglass exhibit low mechanical properties, especially during in-vitro immersion [185,186]. Although a large number of investigations had been performed on these composites (with different bioglass compositions and manufacturing processes), the majority of studies were mainly focused on the characterization of cytotoxicity, biocompatibility, cell adhesion, or bioactivity.

Some studies address the influence of bioglass composition on the properties of a composite. For example, by modifying the BG composition it is possible to reduce their reactivity (thus delaying their bioactivity) and therefore limit the degradation of the polymer matrix. According to this approach, composites based on bioglass fillers containing less sodium show better mechanical properties while ensuring the bioactive properties.

Unfortunately, when it comes to optimizing the manufacturing processes, there are few studies dealing with a deeply characterization and prediction the final mechanical and morphological properties.

For most polymer processing techniques, the viscosity of the molten material needs to be in a certain range to ensure a good processability and the suitable properties of the final product. A rheological study provides information regarding the filling properties and flow of biocomposites. Indeed, rheology can be a valuable tool to improve quality control, process efficiency, product quality or reduce energy costs. For example, changes in the chemical composition of materials at high temperature and their effect on the manufacturing process and on the final product, can be clarified. Hence, rheology can contribute to establish the optimal parameters of a manufacturing process depending on the raw material used (poly(D,L-lactide) (PDLLA)/BG) to meet the complex specifications of medical devices.

The main advantages of additive manufacturing techniques are the design of scaffolds with complex internal structure. However, in the case of composites based on polyester, the mechanical properties of these systems are significantly lower to those of bone, and cannot be used for load-bearing applications. Hence, for these biocomposites, obtaining a low porosity rate is the only way to ensure systems with good mechanical properties. 

Functionally graded materials (FGM) have several advantages, such as the ability to mimic the porosity of the bone, the control and increase of the mechanical resistance or to provide an antibacterial or anti-inflammatory effect. As explained earlier, at high temperatures, the polymer hydrolysis catalyzed by the presence of bioglass, accelerates the polymer chain-scission. Moreover, the in-vitro studies have shown that, when in contact with biological fluids, bioglass accelerates the composite degradation (in particular the mechanical properties). It seems that the use of FGM is the only solution for 3D material extrusion techniques to guarantee the functionality of medical devices for a sufficiently lifetime. 

Besides, to avoid problems of matrix degradation due to the chemical reaction between the matrix and the filler at high temperature, several authors chose to use solvent-based processes. The main limitation of these manufacturing techniques, however, is the raised probability of causing toxic reactions during implantation, as some chemical residue can remain in the final product. No previous study has succeeded to bypass the degradation effect of high temperatures applied during thermal process techniques like extrusion, injection molding, or 3D printing extrusion. Consequently, the processing of resorbable load-bearing devices is an actual challenge which have to address two points:

1. Controlling resorbability induces a robust manufacturing process for bioactive composites.
2. Maintaining sufficient functional mechanical properties of composites during implantation.
3. The commercial devices for bone regeneration are mostly made of ceramic or metallic component, with the mechanical restrictions inherent to these systems (stress-shielding...). The objective of this review was to present the state of the art on the manufacturing processes for promising resorbable composites based on polymer matrix and bioactive glass fillers, to promote osteo-induction. Most of the works presented focuses on academic developments of scaffolds. It is very difficult to compare the intrinsic performances associated with each process because polymer, filler and their ratio are different from one to other process. However, it is obvious that a scaffold can never be used as load bearing device. Thus, the ultimate challenge is to produce hydride load bearing systems as previously detailed in this last section.

## Figures and Tables

**Table 3:** Advantages and disadvantages of additive manufacturing processes and commonly used materials in tissue engineering.

Technique	Advantages	Limitations	References
Fused deposition modeling (FDM)	Multi-material printing, low cost, complex geometries, good strength	Anisotropy, porosity, Easy to block nozzles	[136]
Direct Pellet multi Extrusion Printing	Avoid filament fabrication, multi-material printing, complex geometries, good strength	Anisotropy, porosity, Easy to block nozzles	[137]
Robocasting	Non-thermal degradation, high filled systems	low mechanical properties, porosity, residual solvent products, long time fabrication	[138]
Electrospinning and Melt Electrospinning Writing (MEW)	Interconnected pores, good strength, high specific surface, uniform and aligned fibers, precisely controllable structure	Residual solvent products, high voltage apparatus, limited geometry	[139,140]
Selective laser sintering (SLS)	Design flexibility, good resolution, low material waste, No need for support structure	High cost, long printing time, residual stress, need post-processing, expensive, powdery surface	[141]
Stereolithography (SLA)	Smooth printing surface, good strength	High cost, need of photosensible resins, additional step to eliminate non-cured polymer remining in the scaffold	[139]

**Table 4:** Principal properties of FDM investigations. With * the compression (not filled cells mean no data).

Composite	Modulus (MPa)	Strength (MPa)	Strain at Break (%)	Porosity	Pore Size (µm)	Particle Size (µm)	Content (wt%)	Technique	By
PCL/S53P4	148–157 *			30–40	400	50	10	FDM	[143]
PDLA				48	414			FDM	[144]
PDLA/HA				48	412	50	10		
PDLA/45S5				48	396	38–75	10		
PDLGA	466 *	19.5 *						FDM	[146]
PDLGA/ dilatom-BG	186 *	10.7 *				5–80	1		
PDLGA/ dilatom-BG	264 *	11.3 *				5–80	5		

**Table 5:** Principal properties of Robocasting investigations. With * the compression, ** tensile and *** flexural modulus/strength (not filled cells mean no data).

Composite	Modulus (MPa)	Strength (MPa)	Strain at Break (%)	Porosity	Pore Size (µm)	Particle Size (µm)	Content (wt%)	Technique	By
PCL/BG					500	3 [161]	75	Robocasting	[147]
PCL/mesoBG	4.3 *			75	190/0.005	<25	35.5	Robocasting	[134]
PCL/mesoBG/Na1	2.2 *			84	190/10/0.005	<25	35.5		
PCL/mesoBG Na0.5	3.3 *				190/10/0.005	<25	35.5		
PCL/13-93B3					100–300	20	50	Robocasting	[133]
PDLLA	24.5 **	1 **					0	Robocasting	[148]
PDLLA/BG-B3	22.2 **	1.1 **				20	33		
PDLLA/BG-B3	25.8 **	2.2 **				20	50		
PDLA-PGA	27 *				75–165			Robocasting	[149]
PDLA-PGA/G5	44.2 *				45–165	<40	50		
PCL	75 *	4.2 *		50	370		0	Robocasting	[151]
PCL/BG-45S5	50 *	3.7 *		50	370		10		
PCL/BG-45S5	43 *	3.2 *		50	370		20		
ICIE16						15.8	100	Robocasting	[152]
13–93						10.8	100		
PSrBG						12.5	100		
45S5		13 *	3.8 *	60		1–10	100	Robocasting	[155]
PCL/13-93B3	17 *	90 */20 ***						Robocasting	[158]
PLA/13-93	18 *	105 */22 ***							

**Table 6:** Principal properties of electrospinning investigations. With ** tensile (not filled cells mean no data).

Composite	Modulus (MPa)	Strength (MPa)	Strain at Break (%)	Porosity	Pore Size (µm)	Particle Size (µm)	Content (wt%)	Technique	By
PCL		2.3 **	119			<100 nm	0	Electrospinning	[160]
PCL/nBG		3 **	103			<100 nm	5		
PCL/nBG		3.3 **	108			<100 nm	10		
PCL/nBG		3.4 **	96			<100 nm	15		
PCL/nBG		2.7 **	70			<100 nm	20		
PLLA/BG	35 **	7 **	15		0.1	2	35 vol%	Electrospinning	[161]
PLLA	20 **	11 **	20		0.1	-	-		
PCL	4.61 **	20 **	167				0	Electrospinning	[87]
PCL/nBG	3.7 **	15 **	143			0.032	0.75		
PCL/nBG/DP	6.5 **	21 **	112			0.032	0.75		
PLA/BG	4 **	0.05 **	80			0.027	20	Electrospinning	[162]
PLA/BG/MgO	4 **	0.03 **	30			0.027	10		

**Table 7:** Principal properties of SLS investigations. With * the compression, ** tensile and *** flexural modulus/strength. (not filled cells mean no data).

Composite	Modulus (MPa)	Strength (MPa)	Strain at Break (%)	Porosity	Pore Size (µm)	Particle Size (µm)	Content (wt%)	Technique	By
PDLLA	68.07 ***	1 ***	22 ***			150–300	0	SLS	[167]
PDLLA/58S	79 ***	1.7 ***	6 ***			150–300/12.5	10		
PDLLA/58S	21 ***	0.6 ***	4 ***			150–300/12.5	20		
PDLLA/58S	10 ***	0.2 ***	3 ***			150–300/12.5	30		
PCL	86 **	4.4 **	10			50		SLS	[168]
PCL/β-TCP	124 **	2 **	3			50/3–5	10		
PCL/β-TCP	117 **	1.2 **	1.7			50/3–5	50		
PLDLA	68 ***	3.7 ***	23 ***	38	200			SLS	[169]
PLDLA/58S	79 ***	2.4 ***	6.9 ***	26	200	12.15	10		
PLDLA/58S	21 ***	0.5 ***	4 ***	27	200	12.15	20		
PLDLA/58S	10 ***	0.3 ***	7.4 ***	30	200	12.15	30		
PLLA	1800 *	20.8 *			400			SLS	[172]
PLLA/mBG	3100 *	50.2 *			400	0.5	5		
PLLA/p-mBG	3600 *	62.9 *			400	0.5	5		
PGPL-DEX	95 *	6.2 *		40.4	450			SLS	[173]
PGPL-DEX/mBG	230 *	12.1 *		40.4	450		15		
PGPL-DEX/ p-mBG	270 *	17.5 *		40.4	450		15		
PLA	890 *	10.5 *			400			SLS	[174]
PLLA/mBG	1100 *	15.1 *			400	0.4	4		
PLLA/Ag-mBG	1180 *	15.9 *			400	0.4	4		
SA/13-93		20.4 *		50.3	300–800	42.08	100	SLS	[175]
SA/13-93		41 *		40	300–800	16	100	SLS	[176]

**Table 8:** Principal properties of SLS investigations. With * the compression, ** tensile and *** flexural modulus/strength (not filled cells mean no data).

Composite	Modulus (MPa)	Strength (MPa)	Strain at Break (%)	Porosity	Pore Size (µm)	Particle Size (µm)	Content (wt%)	Technique	By
PCL	1.4 *			77	594	<45	5	SLA	[178]
PCL/S53P4	2.4 *			75	555	<45	10		
PCL/S53P4	2.4 *			70	517	<45	15		
PCL/S53P4	3.4 *			63	476	<45	20		
Photopolymer/45S5		3.2 **		66	870	5		SLA	[179]
Photopolymer/45S5		4.9 **		65	700	5			
Photopolymer/45S5		6.7 **		66	550	5			
Photopolymer/pre-sintered-45S5		8.5 **		63	550	5			
45S5 BG		40 ***		50			100	SLA	[182]
45S5 BG		21.9 *		50			100	SLA	[181]
45S5 BG		124 ***				2	100	SLA	[183]

## Data Availability

Not applicable.
